# P36 Improving AMS programmes for paediatric services via a shared post working collaboratively across five hospital trusts within South Yorkshire

**DOI:** 10.1093/jacamr/dlae136.040

**Published:** 2024-08-23

**Authors:** Charlotte Fuller, Neil Leavesley, Fran Joslin, Fiona Shackley, Lucy Hinds, Caroline Kerrison

**Affiliations:** Sheffield Children’s NHS Foundation Trust, Sheffield, UK; Sheffield Children’s NHS Foundation Trust, Sheffield, UK; Sheffield Children’s NHS Foundation Trust, Sheffield, UK; Sheffield Children’s NHS Foundation Trust, Sheffield, UK; Sheffield Children’s NHS Foundation Trust, Sheffield, UK; Sheffield Children’s NHS Foundation Trust, Sheffield, UK

## Abstract

**Methods:**

This project is run by a paediatric AMS leadership fellow, funded 50% by NHS England and 50% by South Yorkshire ICS, as an out of training programme experience (OOPE) through the Future Leaders Programme. This 12 month post allocates 0.3 FTE to embedding AMS principles and practices into the routine care of children accessing Barnsley, Chesterfield, Doncaster, Sheffield and Rotherham hospital trusts. A service evaluation was first undertaken to identify compliance of hospital AMS programmes with national and international guidance, followed by network formation of 32 healthcare professionals across the South Yorkshire region, including paediatricians, microbiologists and pharmacists. A regional antimicrobial prescribing monitoring and feedback system was launched, providing bi-monthly central analysis and reporting of individual trust and regional data, enabling trusts to meet monitoring requirements with only data collection as local time spent. A standardized regional education programme has been designed, with the curriculum agreed across the network. Focusing on attitudes towards responsibility, AMS principles and human factors in prescribing, this has been delivered face-to-face to paediatric prescribers in each hospital, with pre- and post-session confidence scoring in four domains.

**Results:**

Baseline service evaluation revealed inconsistencies in AMS programmes between trusts, with only 53% of quality indicators fully met in paediatric services, compared with 73% in adults. The predominant reason reported for suboptimal compliance was staff shortages within hospital AMS teams. Since project inception (8 months), quality indicator compliance within paediatric services across South Yorkshire has increased from 53% to 65%, closing the gap between adult and paediatric AMS support. Three rounds of antimicrobial prescribing data have been collected. For prescriber confidence, the challenging prescriptions domain scored lowest. The regional education programme has increased overall prescriber confidence scores from 71.3% to 89%. Further regional AMS interventions, facilitated by the collaborative network, are in progress including regional audits and a regional antibiotic guideline.

**Conclusions:**

Forming an AMS-focused regional collaborative network has increased awareness of services and barriers across the region, allowed for coordinated interventions familiar to rotating trainees, and facilitated the sharing of resources and learning. This approach has reduced the workload of local AMS teams by addressing the barrier of staff shortages, alongside increasing coverage of AMS programmes to paediatric services. ICBs across the UK could choose to fund similar posts dedicated to the co-ordination of AMS programmes across regions, maximizing efficiency in a climate of ongoing workforce challenges.

